# Sepsis among Neonates Admitted to a Neonatal Intensive Care Unit in a Tertiary Care Centre

**DOI:** 10.31729/jnma.8431

**Published:** 2024-02-29

**Authors:** Kanchan Devkota, Piush Kanodia, Bibek Joshi

**Affiliations:** 1 Nepalgunj Medical College and Teaching Hospital, Nepalgunj, Banke, Nepal; 2 Department of Pediatrics, Nepalgunj Medical College and Teaching Hospital, Nepalgunj, Banke, Nepal

**Keywords:** *neonate*, *neonatal sepsis*, *prematurity*, *prevalence*

## Abstract

**Introduction:**

Neonatal sepsis is a condition that carries a high risk for mortality as neonates rapidly transition to extra-uterine life and are subjected to various risk factors. Sepsis prevalence can be reduced by good antenatal care, early detection and treatment of risk factors. The study aimed to find out the prevalence of sepsis among neonates admitted to a neonatal intensive care unit in a tertiary care centre.

**Methods:**

This is a descriptive cross-sectional study conducted among neonates admitted to the neonatal care unit of a tertiary care centre after obtaining ethical approval from the Institutional Review Committee. Data of patients admitted from 12 December 2022 to 30 June 2023 was collected from hospital records. Symptomatic patients admitted to the neonatal intensive care unit were included and those with incomplete data were excluded from the study. A convenience sampling method was used. The point estimate was calculated at a 95% Confidence Interval.

**Results:**

Among 379 neonates, the prevalence of sepsis was 138 (36.41%) (28.38-44.44, 95% Confidence Interval). A total of 98 (71.01%) had early-onset neonatal sepsis and 40 (28.99%) had late-onset neonatal sepsis.

**Conclusions:**

The prevalence of neonatal sepsis was found to be lower than other studies done in similar settings.

## INTRODUCTION

Neonatal Sepsis is a condition that carries a high risk for mortality in neonates. From acting as a unit with their mothers to initiating their defence mechanisms, neonates are subjected to various risk factors. The 2016 NDHS shows that the neonatal mortality rate (NMR) has moved from 33 per 1,000 live births to 21 per 1000.^1^

The pattern of neonatal diseases varies with variations in geography. In developed countries, the main cause of morbidity and mortality in the neonatal period is congenital abnormalities which are mostly non-preventable, but in developing countries causes such as infections, birth asphyxia and pneumonia predominate.^2^ Most of these are preventable by good antenatal care, early detection and treatment.^3^ To improve neonatal survival, appropriate interventions should be directed towards illnesses and their prevention.^4^

This study aimed to find out the prevalence of sepsis among neonates admitted to a neonatal intensive care unit in a tertiary care centre.

## METHODS

This descriptive cross-sectional study was conducted among patients admitted to the Neonatal Intensive Care Unit, Nepalgunj Medical College and Teaching Hospital, Nepalgunj, Banke, Nepal. Ethical approval was obtained from the Institutional Review Committee of the same institute (Reference number: NGMC-IRC/079/080-34). Data was collected from 12 December 2022 to 30 June 2023 from inpatient records at the hospital record system. Neonates admitted during study period is included in the study. Those with missing data were excluded from study. A convenience sampling method was used. The sample size was calculated by using the following formula:


$$\begin{array}{ll} \text{n} & ={\text{Z}}^{2}\times \frac{\text{p}\times \text{q}}{{\text{e}}^{\text{2}}}\\ & =1.96^{2}\times \frac{0.50\times 0.50}{0.06^{2}}\\ & =267 \end{array}$$

Where,

n = minimum required sample size
Z = 1.96 at 95 % Confidence Interval (CI)
p = prevalence taken as 50% for maximum sample size
q = 1-p
e = margin of error, 6%

The minimum required sample size was 267. However, the final sample size taken was 379.

Prematurity was diagnosed according to the WHO definition of live-born neonates delivered before 37 weeks of gestation and also by incorporating Ballard scoring. Birth weight 15002500 grams was categorized as low birth weight, 1000-1500 grams was categorized as very low birth weight and <1000 that was classified as extremely low birth weight. Neonatal sepsis was diagnosed based on clinical profile, blood culture and septic screening, onset in <72 hours was classified as early onset neonatal sepsis (EONNS) and >72 hours was late-onset neonatal sepsis (LONNS).^3^

Data were entered in Microsoft Excel 2016 and analyzed using IBM SPSS Statistics version 25.0. The point estimate was calculated at a 95% CI.

## RESULTS

Among 379 patients, the prevalence of neonatal sepsis was 138 (36.41%) (28.38-44.44, 95% CI). A total of 98 (71.01%) had early-onset neonatal sepsis and 40 (28.99%) had late-onset neonatal sepsis. The majority of neonates admitted to the NICU were male 85 (61.59%) and 53 (38.41%) were female. The most common mode of delivery was spontaneous vaginal delivery 86 (62.32%) (Table 1).

**Table 1:** General characteristics sepsis of neonates with (n = 138).

Characteristics	n (%)
**Gender**	
Male	85 (61.59)
Female	53 (38.41)
**Mode of delivery**	
Spontaneous vaginal	86 (62.32)
Cesarean section	52 (37.68)
**Gestational age at birth**	
Preterm	41 (29.71)
Term	70 (50.72)
Post-term	27 (19.57)
**Birth weight**	
Normal	78 (56.52)
Low birth weight	35 (25.36)
Very low birth weight	25 (18.12)

**Figure 1:**
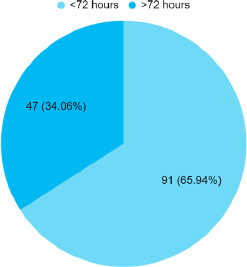
Hours of life of neonates with sepsis (n = 138).

A total of 91 (65.94%) neonates presented within 72 hours of life while 47 (34.06%) presented after 72 hours of life (Figure 1).

## DISCUSSION

In our study among 379 patients, the prevalence of neonatal sepsis was 138 (36.41%). A similar study listed major causes of admission of neonates as sepsis with prevalence of 36.9%.^3^ The leading cause of death among neonates in Nepal was found to be neonatal sepsis followed by prematurity, low birth weight and birth asphyxia.^5^ One of the significant causes of neonatal morbidity and mortality especially in preterm, low birth weight infants is neonatal sepsis.^6^

Our study shows, the majority of neonates admitted to NICU were male, 85 (61.59%) while 53 (38.41%) were female. A total of 86 (62.32%) were delivered through spontaneous vaginal delivery and 52 (37.68%) by cesarean section. A total of 70 (50.72%) were term deliveries, 41 (29.71%) were delivered pre-term and 27 (19.57%) were post-term deliveries. A similar study showed similar demographics of 57.7% males and 42.3% females with the majority being term gestation 89.2% and 10.8% being pre-term.^3^ The majority were born via spontaneous vaginal delivery (55%) while 39.4% were born via cesarean section.^3^ Another research showed male predominance with 65.5% males and 34.4% females.^7^ Male predominance may be because males are given more attention by caregivers and brought to seek health services more often in our country.^8^

The most common predisposing factor for susceptibility to infection in neonates includes premature birth and low birth weight.^9^ In this study, the majority of admissions were done within 72 hours of life 98 (71.01%) while some were after 72 hours of life 40 (28.99%). A total of 35 (25.36%) neonates were within the range of normal birth weight whereas, 35 (25.36%) were of low birth weight and 25 (18.12%) had very low birth weight. Another study shows 75.6% of neonates within the normal weight range and 24.4% below the range.^10^ Likewise, 65.58% admissions within 72 hours of life and 34.41% after 72 hours, likewise 65.11% were within the normal range of weight while 34.89% were below the range in a similar study.^11^ There is a 3-10-fold higher incidence of infection seen in preterm neonates than in full-term normal birth weight neonates.^12^

There are also some limitations of this study as it is a single-centered study and might not be generalized to the entire population.

## CONCLUSIONS

The prevalence of neonatal sepsis among neonates admitted to the neonatal intensive care unit was lower than other studies done in similar settings. Further studies involving multicentered research are needed to enhance the generalizability of findings and better understand the factors contributing to the neonatal sepsis in the neonates.
